# Medical publishing and the threat of predatory journals

**DOI:** 10.1016/j.ijwd.2016.08.002

**Published:** 2016-09-23

**Authors:** Jeffrey Beall

**Affiliations:** University of Colorado Denver

## Introduction

You have probably received spam e-mail solicitations from previously unknown publishers inviting you to submit a manuscript to one of their journals, join an editorial board, or, perhaps, complete an ad hoc peer review of a scholarly manuscript. In fact, if you are like most researchers in the biomedical sciences, you have probably received such e-mails on a daily basis. Most of these spam e-mails come from what I have termed *predatory publishers*: low-quality publishers that want to exploit your expertise and your need to publish. Along with other academic librarians, I have been tracking predatory publishers and monitoring their evolution. The aim of my work has been to help researchers avoid becoming victims of these exploitative and dishonest publishers and to show how they are threatening research. In this article, I will describe predatory publishers, identify how they operate and hurt researchers and science, and show dermatology researchers how best to avoid them.

The early 2000s saw the emergence of the open-access movement, a social movement that argued for and promoted the transition to open-access publishing for academic journals. Under the successful, subscription journal publishing model, library subscriptions were the chief method to finance the publication of scholarly research. Open-access introduced a new method of financing journals in the form of author fees. In most cases, with the use of this publishing method, journals are freely accessible, and the publishing costs are covered by fees charged to authors upon acceptance of their articles for publication.

In theory, the idea is great: no-cost access frees academic and medical libraries from having to pay subscription charges, and published articles are accessible to anyone, anywhere, with internet access. Indeed, there are many open-access journals using the author-pays model that are ethical and successful. Although it does have weaknesses, the model itself is not the problem. The problem is the abuse of the author-pays model for profit, leading to the profusion of predatory publishers. Thus, while the open-access publishing model was born with noble intentions, many such initiatives have unintended, negative consequences, and open-access publishing is no exception.

In this case, the downside is the built-in conflict of interest inherent in the open-access publishing model. Publishers who employ the model generate increased revenue if they publish more papers. This is great for the publisher but bad for science. The conflict of interest is in stark contrast to the demands of peer review, which, if performed honestly, often results in papers being rejected for publication. Marginal publishers realized this profitable weakness soon after the open-access movement started and began to seek as many manuscripts as possible for publication to maximize their income. Realizing this, I coined the term *predatory publisher* (Beall, 2010).

## Predatory medical publishers

Frequently, when new predatory, open-access medical publishers appear, they launch with a large number of journals. Typically, they use a template to create a home page for each journal and try to have one journal for each major medical specialty. As a result, many low-quality and predatory open-access publishers include a dermatology journal among their titles.

The predatory publishers know that medical researchers often have research grants and that they frequently use this funding to pay author fees. That is why medical researchers are targeted by so many spam e-mails: predatory publishers want a share of their grant money.

Predatory publishers operate like counterfeit scholarly publishers. Many pretend to be scholarly societies, associations, or institutes when in reality, they are merely a privately held microbusiness, often operated from a dwelling. Many hide their business locations, or use virtual office companies to make it appear as if they are based in an Anglophone country. They promise a fast publishing process, and some even optionally charge a separate fee for an expedited review process of a week or two. Some add researchers' names and university affiliations to their editorial boards without the permission or knowledge of these researchers. They try to exploit the names and affiliations to attract article submissions, which is their bread and butter.

For much of its history, science has relied on a gentleman's agreement (Beninger et al., 2016) to govern the quality control of the communication of science. As part of this tacit bond, authors agreed to carry out only honest and ethical research, and journals agreed to manage peer review fairly and honestly.

Both agreed to employ peer review and the scientific consensus to demarcate authentic science from pseudo-science to maintain the academic record's integrity. Thus, there have been scientific journals focusing on astronomy but none on astrology. No human activity works perfectly all the time, and peer review is no exception. However, the gentleman’s agreement has completely broken down with the advent of predatory journals, which can be used to promulgate any scientific thesis all while bearing the window dressing of science. This breakdown is harming science.

## The damage they cause

Predatory and low-quality journals enable the publication of pseudo-, activist, and conspiracy-theory science. Medical science has been particularly hit hard, with journals now devoted to unscientific medicine such as Ayurveda and homeopathy. Activist science seeks to promote a political or social cause, such as denying anthropogenic global warming. Conspiracy theorists have used open-access journals to promote the chemtrail conspiracy theory, namely that governments use airplanes to spray chemicals into the atmosphere.

Some have used pay-to-publish journals to falsely prove the efficacy of medicines they have developed. For example, several articles have been published in predatory journals promoting an unapproved drug called GcMAF. It is clear that the authors of those articles exploited the easy or automatic article acceptance in the journals where the articles were published. The Belgian Anticancer Fund has done work to inform the public about GcMAF (Anticancer Fund, 2015).

Publishing in a predatory journal or voluntarily serving on the editorial board of one can hurt researchers' careers. When such articles or editorial board services are recorded on one’s curriculum vitae, it can actually hurt a researcher’s chances of earning promotion and tenure. External reviewers may take note of one's publication in easy-acceptance journals, penning evaluations that hurt a career rather than help it (Glick, 2016).

Author fees may prevent some researchers without grants from publishing in predatory journals (Tzarnas and Tzarnas, 2015), especially emeritus faculty and researchers in middle-income countries where both grants and fee waivers are rare. A recent study pegged the average article processing charge for an open-access journal in North America at slightly below $2,000 (Solomon and Björk, 2016). It is apparent that in some cases, more prestigious open-access journals will charge higher fees because the demand to publish in these journals is greater.

Easy acceptance, open-access journals also hurt academic evaluations. Some researchers exploit the quick, easy, and cheap publishing process that so many predatory open-access journals now offer. Too many academic evaluation systems have not kept up with the changes in scholarly publishing and rely too much on academic evaluation by means of counting one's number of publications. Such systems enable dishonest researchers to easily outperform their honest counterparts, who submit their work to journals where the bona fide peer review process takes longer and the risk of eventual rejection is higher. This miscarriage of academic evaluations results in a deep resentment among honest researchers toward those who publish with easy-acceptance journals.

## Avoiding predatory journals

In their article, "Caught in the trap: The allure of deceptive publishers," nursing researchers Nicoll and Chinn (2015) describe several ways that scholars rationalize publishing in predatory journals. One situation involves authors who experience multiple rejections for an article submitted to several journals. Such authors are sometimes successfully lured into submitting the manuscript to a low-quality or predatory journal, just to see it published.

## Conclusion

Researchers should avoid the temptation to submit their work to easy-acceptance journals. The long-term damage of such a decision can hurt one's career and stigmatize research carried out later. Stick to known publishers and journals, and be skeptical of any publishing-related solicitation you receive through e-mail (Beall, 2016).

No research is more important to humans than medical research, and it deserves to be published in top-quality journals that professionally manage peer review and add value through copyediting and promotion of published articles. Aim for the top. Do the best possible research and share it with colleagues by publishing it in the top journals in your field.
